# Yes! Maximizers Maximize Almost Everything: The Decision-Making Style Is Consistent in Different Decision Domains

**DOI:** 10.3389/fpsyg.2021.663064

**Published:** 2021-07-21

**Authors:** Emilio Moyano-Díaz, Rodolfo Mendoza-Llanos

**Affiliations:** ^1^Faculty of Psychology, University of Talca, Talca, Chile; ^2^Department of Social Science, School of Psychology, University of Bío-Bío, Chillán, Chile

**Keywords:** satisficing, services, life decision, health, finances, consumption, maximization

## Abstract

The decision-making literature distinguishes one maximizing style from another satisficing decision-making style, but it is unknown whether these styles remain stable or are variable depending on the occasion. One way to approach it is to verify eventual generalization of these styles in behavior of people in different decision domains. Some incipient results with University students from the United States and Austria suggest that these styles would remain in three different domains. However, it is unknown if this is the case in adults, other cultures, or vital areas of great relevance, such as health and personal finances. The objective here is to identify if Chilean Latin American participants of different sex and age maintain their decision-making style in five different decision domains. The sample was 343 volunteers, 52.6% men, from two regions of central-southern Chile (Maule and Ñuble), aged between 20 and 90 years (*M* = 45.47; *SD* = 16.05), who answered the Maximization Tendency Scale, and 45 items corresponding to five different decision domains: health, life decision, finances, services and experiences, and consumer's good. An apparent coherence of decision-making style—maximizing and satisficing—was obtained in the five domains. The health domain stands out for being the one in which it is maximized and with greater internal homogeneity.

## Introduction

Simon's (1955) theory of limited rationality established that searching for the optimal option when deciding (maximizing) was limited because human beings cannot capture and process the enormous information available about the often very numerous possible options when deciding. Maximizing implies high efforts to capture and process information until the optimal choice is reached, which would be a learned or unnatural style of deciding. Thus, it would be more of a human nature to face decisions by satisficing, that is, by choosing only one good enough option.

In a society characterized by a wide range of consumer products, goods, and services, consumers are urged to maximize their purchasing decisions (Moyano-Díaz and Mendoza-Llanos, 2020). The process of maximizing may initially be attracted to the decision-maker, but it becomes difficult, mainly because of the overloaded information, which means searching, analyzing, and weighing it exhaustively (Carnevale et al., 2011, Iyengar and Lepper, 2000).

In this context, the evidence shows that those who exercise a maximizing decision style are more likely to experience negative emotional consequences than those who simply satisfy (Schwartz et al., 2002; Abbe et al., 2003; Iyengar et al., 2006; Pelusi, 2007; Dar-Nimrod et al., 2009; Bin Rim et al., 2011; Purvis et al., 2011; Moyano-Díaz et al., 2013, 2014; Cheek and Schwartz, 2016). However, little research exists on whether people maintain and generalize the same decision-making style in their different operating environments. Hence, it is unknown whether people are consistently maximizing or satisficing in their behavior and how widely. It seems reasonable to accept that there is an alternation in styles depending on the situations they face. On the other hand, it is quite different from deciding where to wash the car than to decide which medical specialist will be better for treating the illness of a child, being more likely in the first case to decide with satisfying style than maximizing.

In studies on maximization, the use of Schwartz's Maximization Scale (Schwartz et al., 2002) to measure it predominates and, occasionally, with dilemmas or stories about the requested decisions (Weaver et al., 2015, for example). However, none of these studies about decisions such as buying a car, clothes, looking for a job, a house, educational offers from universities (Diab et al., 2008), choosing between posters (Sparks et al., 2012), films, different flavors of ice cream (Weaver et al., 2015), sexual behavior options (Caña et al., 2015), and friendships (Newman et al., 2017) seek to verify if the decision-making style is maintained or changed depending on the domain or element of the consumer decision in question. Also, based on the most recent reviews of the concept and several maximization measures (Turner et al., 2012; Misuraca et al., 2015; Cheek and Schwartz, 2016; Moyano-Díaz and Mendoza-Llanos, 2020), it was observed that those studies present critical weaknesses that some authors have referred to as conceptual confusion and undesirable proliferation of instruments for measuring maximization (Cheek and Schwartz, 2016; Misuraca and Fasolo, 2018). This weakness makes it unfeasible to integrate the results into a coherent whole.

Many of these studies use Schwartz's Maximization Scale (Schwartz, 2000; Schwartz et al., 2002), currently questioned by the bias that some of its items refer to a different concept of maximization (high standards) and some others that have become obsolete (Diab et al., 2008; Lai, 2010; Rim et al., 2011; Turner et al., 2012; Dalal et al., 2015; Misuraca et al., 2015). Given the weakness in the definition and measurement of maximization, Cheek and Schwartz (2016) propose to use the Maximizing Tendency Scale (MTS)-7 (Dalal et al., 2015) as the most appropriate measure of maximization until a better one is available.

Misuraca et al. (2015) report that decision-making styles would be maintained in at least three areas: general or non-specific, professional and academic, and consumer. Without prejudice to the work carried out by Weaver et al. (2015) on University students, we believe that the study of Kokkoris (2019) would be the first systematic attempt to directly test the hypothesis that maximizers maximize in a wide range of decision domains, overcoming the deficiencies of previous studies that explored this in a limited number of domains. It conceives maximization as a global tendency in decision making, which is reflected in various decision tasks. To this end, he evaluated the choice of young USA residents for a set of 16 items in a first study (*n* = 78) and 29 items in the second study (Austrian, *n* = 227), grouped into three decision domains: consumer goods (bottled water, food, detergent, clothes, shoes, sunglasses, perfume, furniture, smartphone, laptop, car), experiences and services (gym, movie, book, concert, TV series, restaurant, meal, cafe/bar, drink, hotel room, holiday destination), and life decisions (residence zone, apartment, job, employer, studies, friends, partner).

In his first study, Kokkoris (2019) used the median outcome in MTS to classify 78 participants as maximizers or satisficers, finding significant positive correlations between maximizing style and the 16 items assessed. Separate scores for the three decisional domains showed maximizing correlations with consumer goods (*r* = 0.38, *p* < 0.001), experiences and services (*r* = 0.27, *p* < 0.015), and life decisions (*r* = 0.45, *p* < 0.001). Seeking to identify effect sizes in the three decisional domains, Study 2 was conducted with a larger online sample of 227 Austrian students (84 men, 143 women, 18–55 years old, with an average age of 24.4 years), where positive correlations between the maximizing traits were reported for 22 of the 29 items. He classified them as maximizers and satisficers, using the mean, and grouped the items by domain, showing that the maximizing trait is positively correlated with the domain (*r* = 0.41, *p* < 0.0001). As reported in Study 1, he reports the following correlations between maximization and each of the three domains: consumer goods (*r* = 0.38, *p* < 0.001), experiences and services (*r* = 0.25, *p* < 0.001), and important life decisions (*r* = 0.40, *p* < 0.001).

A meta-analysis and follow-up considering both studies showed that the maximizing feature correlates positively with maximizing in all three decisional domains and that it is maximized significantly less in the experiences and services domain than in the other two. Maximizers do not differ from each other in the domains of consumer goods and life decisions. Thus, participants maximize less in decisions regarding the experiential than in those regarding purchases of the material, and Kokkoris (2019) suggests that future studies should extend the analysis to health and finance domains.

Although they are undoubtedly a contribution, the results of Kokkoris (2019) correspond to samples of young University students from the USA and Austria and open the questions of whether such results are generalizable to the general and adult population, on the one hand, and to cultures of countries less developed, such as Latin Americans, on the other hand. Additionally, it is essential to specify whether gender and age introduce differences in the decision-making style since women increasingly entered the workforce and made independent consumption decisions. On the other hand, health becomes a more critical area as one advances in age (Moyano-Díaz and Mendoza-Llanos, 2021).

In this study, we believe that people have a hierarchy of decision domains and that, based on it, they would maximize those more important domains regardless of the decision-making style. It would be the case of health and finance, especially, where wrong decisions have more severe consequences than in other domains, e.g., iatrogenesis due to a misdiagnosis or incorrect medication or health treatment, economic losses, or financial bankruptcy. Negative consequences for wrong decisions in these domains impact more at-risk adults and older people than younger people. Adults with greater financial autonomy and life span than young people are less likely to recover from the negative consequences of wrong financial decisions, which could even be irrecoverable. If this were true, it would be reasonable to expect that there would be no difference between maximizers and satisficers in that essential decision domain. In this way, when it comes to matters of high importance to people, they always search maximizing regardless of their decisional tendency.

Hence, this general study aimed to identify if Chilean Latin American participants of different sex and ages maintain their decision-making style in five different decision domains. In order to respond to that aim, we proposed three specific objectives in this research:

1. Describe if there are sex and age differences according to the decision domains.
2. Describe if there are differences in the priority (intensity) of maximization between domains.
3. To identify the maximization relationship with five decision domains in the Chilean adult population, including two new, not yet investigated, domains of great importance for the lives of people: finance and health.

## Method

### Participants

A total of 343 adults from two regions of central-southern Chile (Maule and Ñuble) voluntarily participated in the study, making up a non-random sample based on availability or accessibility. The aim was to select a similar number of participants by sex and cover all the age groups from adolescence to old age so that 47.37% were women and their age ranged between 20 and 90 years (*M* = 45.47; *SD* = 16.05).

Regarding the distribution according to the educational level, 30.9% of the participants had completed University education, 17.7% had completed technical education, 36.1% had completed secondary education, and the remaining 14.9% had completed primary education. This question was skipped by 0.3% of participants.

### Instruments

A questionnaire was constructed with sociodemographic items -sex, age, and educational level- and two instruments whose description follows below.

#### Maximizing Tendency Scale

It is a 9-item scale by Diab et al. (2008) to measure the tendency of maximizing and satisficing decisions. This instrument has been used in the USA, and at least in Canada, Austria, and Chile, on participants between the ages of 16 and 81 from various ethnic backgrounds. The nine statements are written in a 5-point Likert response format, where 1 means “strongly disagree” and 5 “strongly agree.” Here, the Spanish version adapted to Chile by Moyano-Díaz and Mendoza-Llanos (2017) is used, with an internal consistency of Cronbach's alpha of 0.74 in working adults and of 0.80 for the present study.

#### Choice of the Best Option by Domains

Participants were presented with a list of 45 items to decide, which were grouped into five decision domains. Four out of these five domains correspond completely to products or consumer goods, while the remaining one domain referred to as life decisions contained some items that other did not have. For each item, participants had to indicate on a 6-point scale to what extent they want to make the best choice in the respective item (6) or if it is enough for them to choose an option that is satisfactory and good enough (1). The five domains correspond to the three proposed by Kokkoris (2019) with 29 items, consumer goods (smartphone, detergent, furniture, laptop, bottled water, clothing, food, shoes, sunglasses, perfume, car); experiences and services (restaurant, cafe/bar, hotel room, holiday destination, movie, book, meal, drink in a bar, TV series, concert, gym); life decisions (studies, job, employer, friends, partner, department, area of residence), and two new domains with 16 items: health (general practitioner, specialist doctor, surgeon, surgery clinic, laboratory for medical examinations, dentist for control, dentist specialist, implant dentist) and finance (request consumer credit, request mortgage loan, request car loan, deposit savings, open bank account, an insurance company, health insurance, pension management), totaling 45 items.

**Figure 1:**
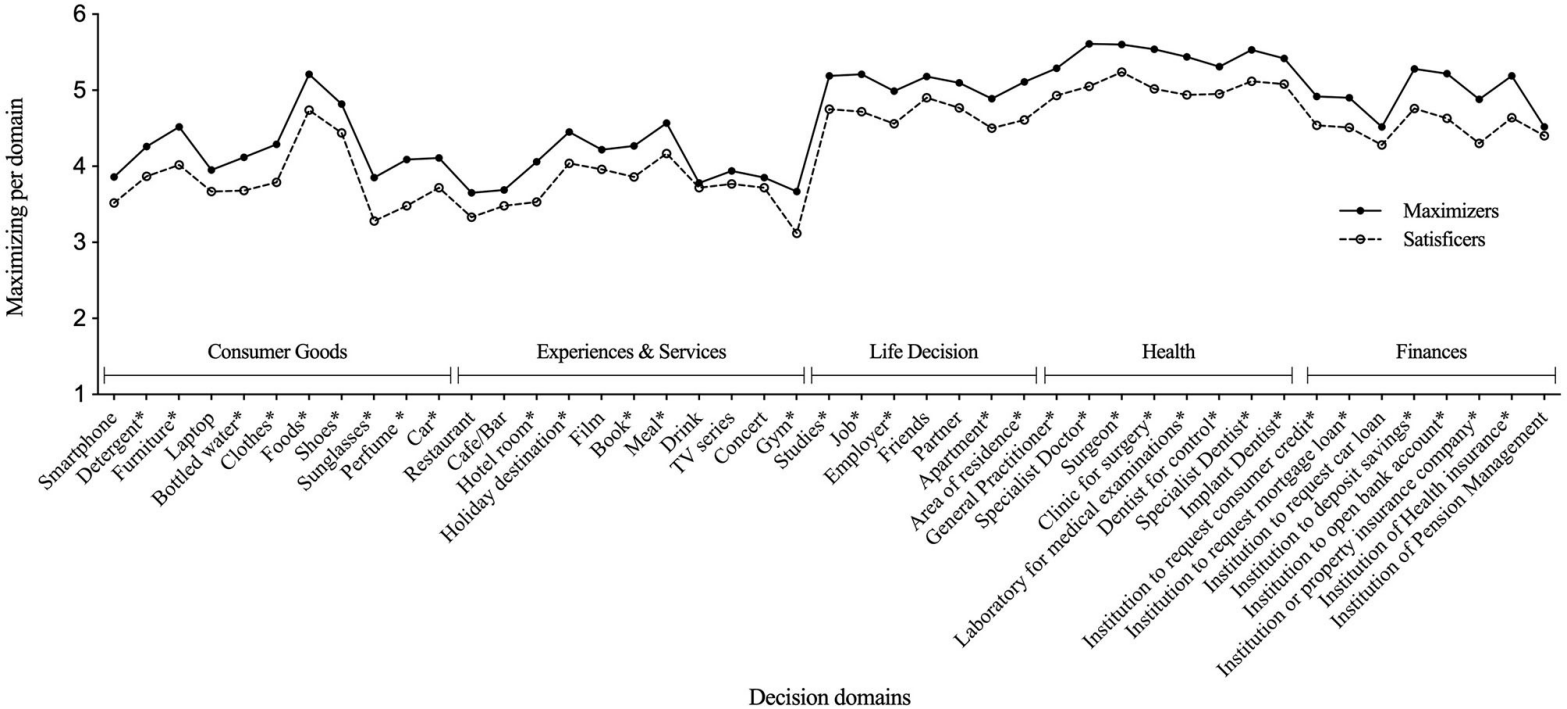
Maximizers' and satisficers' maximizing tendencies in five decision domains. *Differences in the decision domain are statistically significant (*p* < 0.05).

In this study, the reliability for each of the five domains was 0.85, 0.88, 0.79, 0.93, and 0.92, respectively. The English version of all items is shown in Figure 1.

### Procedure

For access to the sample, social networks were used to contact potential study participants. All participants received information on the objectives of the study, emphasizing confidentiality, and voluntary participation in the research. After signing the informed consent, the questionnaire was applied to the study. In order to control for a possible bias in the order of presentation of the items, the items were counterbalanced so that half of the sample participants answered form A of the questionnaire and the other half answered form B. Form A had a sequence of domains: consumer goods (smartphone, detergent, furniture, laptop, bottled water, clothes, food, shoes, sunglasses, perfume, car); experiences and services (restaurant, cafe/bar, hotel room, holiday destination, movie, book, meal, drink in a bar, TV series, concert, gym); life decisions (studies, job, employer, friends, partner, apartment, area of residence); health (general practitioner, specialist doctor, surgeon, surgery clinic, medical examination laboratory, control dentist, specialist dentist, implant dentist); and finance (apply for consumer credit, apply for a mortgage loan, apply for a car loan, deposit savings, open bank account, insurance company, health insurance, pension management). Form B had the sequence of domains in reverse, beginning with finance and ending with consumer goods. In both forms, there were 45 items in total.

Each participant was paid CL$2,500 upon completion of their participation in the study. The Scientific Ethical Committee of the University of Talca approved the study (Folio 2017-08-EM).

### Statistical Analysis

Averages per area and item and comparisons of means were calculated for the two decision-making styles: maximizing and satisficing. In the first place, Pearson's correlations between global decision domains and maximization goals were conducted. Second, a paired-samples *t*-test was conducted to examine the differences between global decision domains. Third, a maximizer group (score above the median, *n* = 156) and a satisficer group (score below the median, *n* = 187) were suggested. A sensitivity power analysis with G*Power 3.1 (Faul et al., 2007) for the *t*-test revealed that the group size generated can reliably detect small effect sizes of *d* = 0.27 (one-tailed) with an alpha level of 0.05 and a power of 0.80. Finally, a *t*-test was conducted to examine the differences between maximizers and satisficers in all different decision domains. Cohen's *d* index is used to assess the differences in effect size, where 0.2 = small, 0.5 = medium, and 0.8 = large (Coe and Merino, 2003; Ledesma et al., 2008). All analyses were performed with JASP 0.13.1 software (JASP Team, 2020).

## Results

Regarding the sociodemographic variables, no statistically significant differences are observed in the decision domains by sex [health (*t*_(340)_ = −0.99; *p* = 0.32), life decisions (*t*_(340)_ = −0.66; *p* = 0.51), finance (*t*_(340)_ = −0.13; *p* = 0.90), consumer goods (*t*_(340)_ = 0.16; *p* = 0.87), and experiences and services (*t*_(340)_ = −1.01; *p* = 0.31)]. Only health correlates with age (*r* = 0.13, *p* = 0.01). The maximizing tendency correlates positively with all decision domains [health (*r* = 0.24, *p* < 0.001), life decisions (*r* = 0.24, *p* < 0.001), finance (*r* = 0.19, *p* < 0.001), consumer goods (*r* = 0.29, *p* < 0.001), and experiences and services (*r* = 0.20, *p* < 0.001)].

The comparison of means for the different domains, health (*M* = 5.23; *SD* = 1.08), life decisions (*M* = 4.87; *SD* = 0.99), finance (*M* = 4.70; *SD* = 1.36), consumer goods (*M* = 4.04; *SD* = 1.06), and experiences and services (*M* = 3.84; *SD* = 1.17), can be seen in Table 1, with an increasing difference in the size of the effects from small to large.

**Table 1:** Comparison of means between different decision domains.

						**95% CI for Mean Difference**		
		**Domains**	***t***	***df***	***p***	**Lower**	**Upper**	***Cohen's d***
Health	–	Life decisions	6.86	342	<0.001	0.26	0.47	0.37
Health	–	Finances	7.71	342	<0.001	0.40	0.67	0.42
Health	–	Consumer goods	18.15	342	<0.001	1.07	1.33	0.98
Health	–	Experiences & services	20.57	342	<0.001	1.26	1.52	1.11
Life decisions	–	Finances	2.44	342	0.02	0.03	0.31	0.13
Life decisions	–	Consumer goods	15.64	342	<0.001	0.73	0.94	0.84
Life decisions	–	Experiences & services	18.62	342	<0.001	0.92	1.14	1.01
Finances	–	Consumer goods	8.32	342	<0.001	0.51	0.82	0.45
Finances	–	Experiences & services	11.48	342	<0.001	0.71	1.00	0.62
Consumer goods	–	Experiences & services	3.58	342	<0.001	0.09	0.30	0.19

*Student's t-test*.

The comparison between maximizers and satisficers by domains (Table 2) shows that maximizers have higher scores on all dimensions than satisficers, with a consistent small effect size.

**Table 2:** Comparison of means by decision domains between maximizers and satisficers.

	**Maximizers**			**Satisficers**					
**Domain**	***N***	***Mean***	***SD***	***N***	***Mean***	***SD***	***t***	***p***	***d***
Consumer goods	156	4.28	1.01	187	3.84	1.06	3.94	<0.001	0.43
Experiences & services	156	4.02	1.19	187	3.70	1.13	2.52	0.01	0.27
Life decisions	156	5.09	0.88	187	4.69	1.05	3.86	<0.001	0.42
Health^*^	156	5.47	0.95	187	5.04	1.14	3.70	<0.001	0.40
Finances^*^	156	4.93	1.21	187	4.51	1.44	2.90	0.01	0.31

* *Levene's test is significant (p < 0.05), suggesting a violation of the equal variance assumption*.

An analysis regarding the distribution of the results for the 45 items evaluated can be seen in Figure 1. The highest values are found in the domains corresponding to health and life decisions, followed very closely by finance, suggesting the existence of certain areas of consumer decision in which people make a special effort to find the optimal option, the best possible decision. In Table 1, the comparison of means confirms significant differences between these two decision domains concerning the other three decision domains.

The distribution of results for the 45 items reflects a very similar trajectory of the curves for satisficers and maximizers, with consistently higher values for the latter, and with a systematic differentiation between the two in 73.3% of the aspects consulted (33 of the 45 decision items, for details, see Appendix 1) grouped into the five different decision domains. Satisficing and maximizing decision styles appear as consistent decisional tendency across these different human performance domains. An analysis based on the number of items in which domains and, in decreasing order, observe differences allows the health domain to be ranked first and above all others in terms of its values and internal homogeneity. All of its items are significantly different for satisficers and maximizers. In the second place are the areas of life decisions, consumer goods, and finance. Finally, experiences and services domain is in the third and last place.

Some aspects to be highlighted concerning the five domains are that satisficers and maximizers are differentiated in all the health domain items, where the latter systematically obtain the highest values. A noteworthy result that reflects the relevance for participants in this decision domain in comparison with the others is that here both satisficers (*M* = 5.04) and maximizers (*M* = 5.47) obtained their highest average. Concerning the life decision domain, we observed the differences in five out of seven items, in which maximizers obtained higher scores than satisficers, and we observed no differences in “friends” and “partner” items. The satisficers obtain their highest value in the item “friends,” while the maximizers in “job.” Regarding the finance domain, the maximizers and satisficers differ in six out of eight items, with higher values for the former, with no differences observed in the aspects “institution to request car loan” and “pension management institution.”

For consumer goods, maximizers show higher scores than satisficers on 9 out of 11 items, but the exception is for the items “smartphone” and “laptop.” Maximizers and satisficers coincide with the item with the highest value: “food.” Finally, in the experience and services domain, it is observed that in 5 out of 11 items, there are differences with higher scores for maximizers in the items “hotel room,” “holiday destination,” “book,” “gym,” and “meal,” and maximizers and satisficers coincide with the last item with the highest value.

## Discussion

This study confirms the consistency of the maximizing and satisficing style in five decision domains, including two essential domains not considered by previous studies (Weaver et al., 2015; Kokkoris, 2019), which are of greater relative importance in the lives of people: health and finance.

Our results also broaden and deepen the previous results by providing initial evidence about age, gender, and cross-cultural generalization. The sample used here corresponds to adults, on the one hand, and the Latin American culture, on the other hand (Chileans), contributing to transcultural validity to the consistency or stability of the two main decisional styles. We should add that the sample of adults used is balanced by sex and by whose results allow us to advance in the transcultural validity of the consistency and differentiation between maximizers and satisficers to five decision domains, showing no differentiation by sex.

Thus, we have verified, following our initial hypothesis, that it is indeed in the health domain where maximizers maximize most, thus realizing that people maintaining their decisional style should differ in terms of the intensity of their maximization according to the importance of the field in question. The health domain somehow evokes threats, and in its extreme to survival, which, makes people put great effort or cognitive work to find the best option in this respect. This is also true of the satisficers who, although being below the maximizers in this health domain, nevertheless obtain their highest scores here also concerning the other four (Table 2). The highest values obtained in the distribution of the 45 total items are found in the domains corresponding to health and life decisions, followed very closely by finance, showing the existence of a hierarchy of decision domains in which those in the first place are those in which people make a special effort to seek the optimal option, the best possible decision. Our results are in the same direction and trend as those reported by Kokkoris (2019) in three different consumer domains that are common to both studies: consumer goods, experiences and services, and life decisions.

Although there was no classification or typification of types of consumer goods here, which could merit further study, some results are convergent between maximizers and satisficers worth highlighting. This is the domain of consumer goods in which maximizers and satisficers coincide in granting the highest importance or maximization to the food item, similarly, for the general domain of health. Thus, food and health are areas of decision and consumption to which people, regardless of their tendency or dominant style of decision, are of the utmost importance. With advances in nutrition and medicine, food is currently more strongly associated with health than before, on the one hand. On the other hand, food represents a value that goes beyond nutritional aspects, becoming a social value. Thus, what we eat differentiates, and eventually “distinguishes,” people and social groups according to income and education so that those who are more educated and have a higher income are more likely to be informed to buy and consume healthy food and stay away from “junk food” or unhealthy.

From a practical point of view, given that food is relevant to both maximizers and satisfiers, both are most likely willing to analyze and weigh the nutritional characteristics of products beyond the purely hedonic ones. Thus, those responsible for advertising will be able to increase the information corresponding to the contribution of food products to the health of consumers with the assurance that this will be taken into account when deciding to buy and consume.

Although the results presented here correspond to a sample of Chileans, the scope of these results could be generalized to other Latin American countries (e.g., Brazil, Argentina, Mexico) that possess development characteristics that motivate young adults to decide by thinking about the cost/benefit ratio (Rivera-Aguilera, 2018), which we understand as maximizing.

Finally, behavioral consistency of two decision styles in different domains has been observed here. This supports the initial proposition of Schwartz et al. (2002) that maximizing and satisficing would be personality traits. Previous results (Misuraca et al., 2015; Kokkoris, 2019), including a recent study on decision-making in situations of uncertainty in military contexts (Shortland et al., 2020) and ours, seem to support this proposition. From a theoretical perspective, we can infer that maximizers will experience other negative emotions in each of these five decision domains investigated in this study, thus reinforcing the relation between maximization and negative emotional consequences (Luan and Li, 2017; Moyano-Díaz and Mendoza-Llanos, 2020). Therefore, future studies should evaluate or measure whether this occurs in these five domains considered here in a differentiated manner. Finally, future research needs to explore how these five decision domains could be affected by the private or public context on maximizing (Luan and Li, 2019) because that could explain the non-difference in some items of this study.

## Data Availability Statement

The raw data supporting the conclusions of this article will be made available by the authors, without undue reservation.

## Ethics Statement

The studies involving human participants were reviewed and approved by Scientific Ethical Committee of the University of Talca. The patients/participants provided their written informed consent to participate in this study.

## Author Contributions

EM-D: preparation of the introduction of the manuscript, data collection, and discussion of the manuscript. RM-L: conception and design of the study, preparation of the manuscript's introduction, data collection, data analysis, and discussion of the manuscript. All authors contributed to the article and approved the submitted version.

## Conflict of Interest

The authors declare that the research was conducted in the absence of any commercial or financial relationships that could be construed as a potential conflict of interest.
