# Supplementary material for: Yes! Maximizers Maximize Almost Everything: The Decision-Making Style Is Consistent in Different Decision Domains
Source: Front Psychol. 2021 Jul 21;12:663064. doi: 10.3389/fpsyg.2021.663064 (PMC8335592; doi:10.3389/fpsyg.2021.663064)
Supplement: Supplementary file 1 [file Table_1.docx]

| Appendix 1. Comparison of the decision style means in five decision domains. | | | | | | | | | | |
| --- | --- | --- | --- | --- | --- | --- | --- | --- | --- | --- |
|  | *Maximizers* | | |  | *Satisficers* | | |  |  |  |
|  | N | *Mean* | *SD* |  | N | *Mean* | *SD* | *t* | *p* | *d* |
| **Consumer Goods** |  |  |  |  |  |  |  |  |  |  |
| Smartphone | 155 | 3.86 | 1.76 |  | 187 | 3.52 | 1.78 | 1.77 | .08 | 0.19 |
| Detergent | 156 | 4.26 | 1.66 |  | 187 | 3.87 | 1.68 | 2.15 | .03 | 0.23 |
| Furniture | 155 | 4.52 | 1.44 |  | 187 | 4.02 | 1.49 | 3.14 | .00 | 0.34 |
| Laptop computer | 156 | 3.95 | 1.66 |  | 187 | 3.67 | 1.79 | 1.46 | .14 | 0.16 |
| Bottled water | 156 | 4.12 | 1.86 |  | 187 | 3.68 | 1.95 | 2.14 | .03 | 0.23 |
| Clothes | 156 | 4.29 | 1.70 |  | 187 | 3.79 | 1.60 | 2.82 | .01 | 0.31 |
| Food* | 155 | **5.21** | 1.36 |  | 187 | **4.74** | 1.47 | 3.04 | .00 | 0.33 |
| Shoes | 156 | 4.82 | 1.53 |  | 186 | 4.44 | 1.56 | 2.26 | .02 | 0.24 |
| Sunglasses | 156 | 3.85 | 1.82 |  | 186 | 3.28 | 1.82 | 2.84 | .00 | 0.31 |
| Perfume | 156 | 4.09 | 1.78 |  | 187 | 3.48 | 1.71 | 3.25 | .00 | 0.35 |
| Car | 155 | 4.11 | 1.75 |  | 187 | 3.72 | 1.61 | 2.16 | .03 | 0.23 |
| **Experiences & Services** |  |  |  |  |  |  |  |  |  |  |
| Restaurant | 156 | 3.65 | 1.77 |  | 187 | 3.33 | 1.74 | 1.69 | .09 | 0.18 |
| Cafe/Bar | 156 | 3.69 | 1.68 |  | 187 | 3.48 | 1.63 | 1.18 | .24 | 0.13 |
| Hotel Room | 156 | 4.06 | 1.70 |  | 187 | 3.53 | 1.69 | 2.88 | .00 | 0.31 |
| Holiday Destination | 156 | 4.45 | 1.54 |  | 187 | 4.04 | 1.67 | 2.35 | .02 | 0.26 |
| Film | 156 | 4.22 | 1.81 |  | 187 | 3.96 | 1.65 | 1.39 | .16 | 0.15 |
| Book | 156 | 4.27 | 1.77 |  | 187 | 3.86 | 1.73 | 2.16 | .03 | 0.23 |
| Meal | 155 | **4.57** | 1.59 |  | 187 | **4.17** | 1.68 | 2.26 | .02 | 0.25 |
| Drink (e.g., in a bar) | 154 | 3.78 | 1.81 |  | 187 | 3.72 | 1.71 | 0.33 | .74 | 0.04 |
| TV series | 156 | 3.94 | 1.79 |  | 186 | 3.77 | 1.68 | 0.89 | .37 | 0.10 |
| Concert (or shows) | 155 | 3.85 | 1.89 |  | 187 | 3.72 | 1.86 | 0.66 | .51 | 0.07 |
| Gymnasium | 156 | 3.67 | 1.83 |  | 186 | 3.12 | 1.77 | 2.79 | .01 | 0.30 |
| **Life Decisions** |  |  |  |  |  |  |  |  |  |  |
| Studies* | 156 | 5.19 | 1.31 |  | 187 | 4.75 | 1.51 | 2.87 | .00 | 0.31 |
| Job | 155 | **5.21** | 1.48 |  | 187 | 4.72 | 1.53 | 2.99 | .00 | 0.32 |
| Employer | 155 | 4.99 | 1.53 |  | 187 | 4.56 | 1.49 | 2.63 | .01 | 0.29 |
| Friends | 156 | 5.18 | 1.44 |  | 187 | **4.90** | 1.44 | 1.76 | .08 | 0.19 |
| Partner | 156 | 5.10 | 1.62 |  | 186 | 4.77 | 1.59 | 1.88 | .06 | 0.20 |
| Department | 156 | 4.89 | 1.53 |  | 187 | 4.50 | 1.55 | 2.35 | .02 | 0.26 |
| Residence Zone | 156 | 5.11 | 1.34 |  | 185 | 4.61 | 1.42 | 3.32 | .00 | 0.36 |
| **Finances** |  |  |  |  |  |  |  |  |  |  |
| Institution to request consumer credit* | 156 | 4.92 | 1.49 |  | 186 | 4.54 | 1.65 | 2.25 | .03 | 0.24 |
| Institution to request mortgage loan* | 156 | 4.90 | 1.59 |  | 186 | 4.51 | 1.77 | 2.17 | .03 | 0.24 |
| Institution to request car loan | 156 | 4.52 | 1.78 |  | 187 | 4.28 | 1.84 | 1.23 | .22 | 0.13 |
| Institution to deposit savings* | 156 | **5.28** | 1.28 |  | 187 | **4.76** | 1.62 | 3.23 | .00 | 0.35 |
| Institution to open bank account* | 156 | 5.22 | 1.28 |  | 187 | 4.63 | 1.71 | 3.57 | < .001 | 0.39 |
| Institution or property insurance company* | 155 | 4.88 | 1.64 |  | 186 | 4.30 | 1.84 | 3.03 | .00 | 0.33 |
| Institution of Health insurance (Private, Govt, insurance company)* | 155 | 5.19 | 1.46 |  | 187 | 4.64 | 1.70 | 3.18 | .00 | 0.35 |
| Institution of Pension Management (AFP) | 156 | 4.52 | 2.00 |  | 187 | 4.40 | 1.89 | 0.59 | .56 | 0.06 |
| **Health** |  |  |  |  |  |  |  |  |  |  |
| General Practitioner | 156 | 5.29 | 1.17 |  | 187 | 4.93 | 1.30 | 2.70 | .01 | 0.29 |
| Specialist Doctor* | 155 | **5.61** | 1.08 |  | 186 | 5.05 | 1.35 | 4.15 | < .001 | 0.45 |
| Surgeon * | 156 | 5.60 | 1.11 |  | 187 | 5.24 | 1.25 | 2.76 | .01 | 0.30 |
| Clinic for surgery* | 156 | 5.54 | 1.09 |  | 186 | 5.02 | 1.39 | 3.83 | < .001 | 0.42 |
| Laboratory for medical examinations* | 156 | 5.44 | 1.12 |  | 187 | 4.94 | 1.34 | 3.72 | < .001 | 0.40 |
| Dentist for control | 156 | 5.31 | 1.27 |  | 187 | 4.95 | 1.40 | 2.48 | .01 | 0.27 |
| Specialist Dentist* | 156 | 5.53 | 1.08 |  | 187 | **5.12** | 1.36 | 3.00 | .00 | 0.33 |
| Implant Dentist* | 155 | 5.42 | 1.22 |  | 187 | 5.08 | 1.44 | 2.33 | .02 | 0.25 |
| Note. For all comparisons, the alternative hypothesis specifies that Maximizers will score higher than Satisficers. * Levene's test is significant (*p* < .05), suggesting a violation of the equal variance assumption | | | | | | | | | | |
